# From “safe” to “brave” spaces: pedagogical practices of *exclusion* to promote *inclusion* within & beyond skateboarding

**DOI:** 10.3389/fspor.2024.1456908

**Published:** 2024-08-14

**Authors:** Robert Petrone, Becky Beal

**Affiliations:** ^1^ Department of Learning, Teaching, and Curriculum, University of Missouri, Columbia, MO, United States; ^2^ Department of Kinesiology, California State University, East Bay, Hayward, CA, United States

**Keywords:** skateboarding, youth, exclusion, inclusion—exclusion, safe spaces, brave spaces pedagogy

## Abstract

Through interviews with key stakeholders within skateboard organizations that explicitly attend to issues of diversity, access, and equity, this article explores pedagogical practices that undergird these organizations' programming for justice. More specifically, this article focuses on the interplay between the implementation of practices of *exclusion* to promote, ultimately, *inclusion*. In theorizing this pedagogical approach, this article discusses how notions of “brave spaces” work in tandem with ideas and practices of cultivating “safe spaces” to work toward social transformation within and beyond skateboarding.

“Do you want to hear more about why we need to have an exclusive space to create an inclusive space?”

Amy^1^, Facilitator with Anyone Can Skate

## Introduction

Although there exists a powerful and long-standing ethic of collaboration, cooperation, and participant-support within skateboarding, there have also always existed tensions and concerns around issues of power, representation, access, equity, diversity, and inclusion (1–4). In recent years these topics have received much more overt and sustained attention due to both increased sociopolitical awareness beyond skateboarding and calls for change within the skateboard community. In fact, Willing and Pappalardo (5), in their examination of cultural, social, and political transformation in skateboarding, argue that skateboarding is currently “experiencing a kind of ‘ethical turn’ or a new ‘ethics of skateboarding,’” which they explain is a growing phenomenon—a “boom” as they describe it—whereby “skaters are no longer forced to ‘shut up and skate’ and instead are actively taking a stance for a range of people and social issues that many have always done, but with more energy and urgency post-2020 following the momentum of the *Black Lives Matter* movement” (p. 2–3).

Situated within this context of an “ethical turn” in skateboarding, this article offers an exploration into the *how* of social transformation in skateboarding: How are skateboarding entities that are focused on inclusion and diversity doing this work? What particular mechanisms and pedagogical practices undergird their attempts? What are their “theories of change” (6)? As scholars of skateboarding interested in such intersections of pedagogy and social transformation, we turned to the expertise, experiences, and perspectives of three stakeholders, each of whom is an accomplished skateboarder and a central figure in a skateboard organization that centers inclusion. In engaging these stakeholders, we sought to understand how the organizations they are linked with go about, in concrete terms, attempting to meet their aims of inclusion and access.

Though our inquiry revealed a robust set of pedagogical practices and theories of change across the organizations, in this article, we focus on one: the interplay between the implementation of practices of *exclusion* to promote, ultimately, *inclusion*. Through our conversations with these stakeholders, we were struck by the myriad ways each organization existed in a seeming paradox whereby they purposefully created a set of exclusive experiences for certain participants to facilitate these participants' knowledge, skills, and affective experiences that might engender their abilities and motivations to facilitate a broader sense of inclusion within and beyond skateboarding.

In teasing out the pedagogical practice of exclusion to create inclusion, we see these findings as augmenting the work of Willing and Pappalardo (5), particularly their discussion of the ways micro-level practices and macro-level relations facilitate, what they call, “ethical togetherness” and “ethical place making” (p. 148). Specifically, our findings offer a window into how pedagogies of exclusion offer opportunities for fostering confidence and belonging, as well as knowledge and skills to name, recognize, and intervene in oppressive power dynamics. In this way, our analysis explores a concrete, micro-level way organizations attempt to disrupt inequities and promote a broader sense of “ethical togetherness” and “ethical place making” within what we theorize as “brave spaces.”

## Brave & safe spaces

In building with these stakeholders and their organizations, we theorize the work they are doing as a conscious and dynamic movement between “safe” and “brave” spaces as part of a broader attempt to reconfigure social relations and places. Though many organizations and entities promoting inclusion within skateboarding use language of “safe spaces,” we, in our analysis and drawing from our own work (7, 8), situate this discussion, too, within the idea of “brave spaces” (9).

Drawing on Arao and Clemens (9), by “brave space,” we mean a space whereby participants realize that courage is needed to challenge deeply embedded ways of being and perceiving, and that this engagement can be risky and generate controversy. This is contrasted to “safe spaces” where facilitators try to encourage difficult conversations by developing community agreements to create conditions of safety or comfort. Researchers have noted that this attempt to create safe spaces often does not promote deep dialogue between people of various backgrounds and viewpoints because participants tend to fall back on their assumptions and back off arguments that challenge their social position. Thus, true dialogue and shifts in perspective are rare within such so-called safe spaces when involving a wide range of people.

In teasing out the interplay between “safe” and “brave” spaces, we recognize that all spaces, all learning and participation—whether at skateparks or schools—are always imbued with power dynamics and practices of inclusion as well as exclusion (10–13). Thus, we see a theorization of brave spaces as being generative in imagining social spaces that not only offer a greater sense of inclusivity but also the recognition that participants may always have to re-negotiate and navigate the terms of participation. In this way, our hope, by illuminating these distinctions, might facilitate more nuanced conceptualizing and enacting practices toward equity, diversity, and inclusion in and beyond skateboarding.

## Methods & highlighted organizations

We interviewed key program organizers from the three organizations highlighted in Table 1 below. One of the interviewees was selected based on previous interactions Becky had with him, and the other two participants came from referrals from other contacts Author #2 had with skateboarding insiders. The central criteria used for selecting participants was that they had a prominent role in a skateboarding organization that had explicit programmatic goals involving inclusion, diversity, equity, and/or justice. The highlighted organizations are located in large, diverse urban areas in the United States.

**Table 1:** Overview of highlighted organizations.

Organization	Format/structure	Aims & practices
Anyone Can Skate	Non-profit organization	Create inclusive skate spaces, especially for girls, women, female, nonbinary, trans, and queer skaters
		Empower people to solve their own community issues
Skate in School	After-school club	Focus on youth who are recent immigrants and refugees
		Skateboarding as a tool for joy, community, personal growth
		Addressing fear “in a controlled space”
Skate Center	For-profit entity	Skateboarding as metaphor for life: “How does what you’re learning on a skateboard influence your day-to-day life?”
		Promotes mindfulness and social-emotional learning
		Emphasis on working with boys around issues of (toxic) masculinity

Each interview lasted between 60 and 90 minutes and used a semi-structured approach. The interviews were designed to evoke discussion about: (1) the interviewee's personal trajectory with skateboarding and involvement with the organization; (2) the overarching aims and goals of the organization; and (3) the mechanisms and pedagogical practices the organization used to work with participants toward its aims, especially related to inclusion and access. Across these topics, the interviews were designed to elicit stories, anecdotes, and specific examples rather than generalities.

Each interview was transcribed and open coded separately by Robert and Becky, with particular attention to pedagogical practices. After each initial round of open coding, the research team met for a collective sense-making session during which codes were compared, collated, and collapsed, as well as theory and related literature applied to deepen analysis. After all interviews were completed, the research team met to look across the data set and generate a set of findings related to pedagogical practices. During that session, the decision was made to focus this manuscript on the use of exclusive “safe” spaces to facilitate inclusive “brave” spaces. This decision was made, in large part, due to the ways this pedagogical practice was implored extensively across each organization, how well each participant discussed and theorized the rationale of this practice, and the telling examples each participant shared to illuminate this practice.

Each of these organizations recognizes and explicitly draws on the idea of skateboarding as a metaphor for life and a vehicle to promote transferable understandings and life lessons beyond being on the board. For instance, Skate in School, which primarily serves newly-arrived immigrant and refugee youth, gives explicit attention to the ways trying and practicing skateboarding offers opportunities, “in a controlled environment,” to deal with fear of being in the new social world of the United States, especially as English Language Learners.

Each organizer talked about how skateboarding can be a means for participants to learn how to navigate fear. These organizers pointed to the idea that when people acknowledge their fear, then they can find strategies to manage it. In turn, this can open emotional space to be connected to community and to their own lifelong learning. One organizer stated: “In skateboarding, you have to learn how to fail. And you're just failing, failing, failing. But then, like, you just kind of have this drive. You just want to keep going. Same thing with schoolwork. It's the same thing, the amount of time and effort that they put into learning that trick on the skateboard. Same thing can be applied back over in the schoolwork, and then we work with it.”

Another organizer commented on the value of navigating through fear with support: “Honestly, it is scary. So, I think, like tapping into that. I think it's like a healthy way to be scared and move through that [and] laugh with your friends.” Relying on others as a strategy for overcoming fear and promoting self-discovery was also common. One of the organization's specific goals was to develop mindfulness through “opening up this social and emotional toolbox within oneself, and then have a new way of understanding what it is that you’re learning on a skateboard.” This was specifically addressed through pedagogical strategies to build trust and communication. For example, a skateboarder is blindfolded and led by another person who can only give verbal cues. The skateboarder must rely on the other person for direction as they navigate the park: “Our purpose for that is bringing up fear, ultimately. Then we utilize that as a talking point about: ‘What are other things like fear and anxiety that you have, that you don't necessarily see?’ Then we try to break all that down, to have an open discussion.”

We note these overarching principles of these organizations because they speak to how, though these entities are ostensibly focused on skateboarding, their true aim is bigger than being on the board; in fact, one of the organizers talked about skateboarding being a “hook” to address social issues beyond skateboarding.

## Findings: establishing exclusion to engender inclusion

Though a seeming paradox, each of the organizations deliberately establishes pockets of *ex*clusivity at different times, to, ultimately, engender *in*clusivity. As Amy from Anyone Can Skate says, “We are creating *exclusive* spaces so that we can create the most *inclusive* spaces.” These purposeful practices of exclusion to create inclusion are rooted in the idea that not all (or for some, not many) spaces are “safe” for participants to, as Amy says, “show up as they are.” She explains that participants, particularly those from already marginalized identities, might feel “invisible” and be “scared to take up space.” She says, “They don't see people that look like them, so they're constantly questioning, ‘Do I belong here? Is this for me?’” Establishing exclusion, then, is meant to promote a more secure sense of belonging: “So what we need to do is create a space where they get to really take up space with something like skateboarding.” (Here, we are using “exclusive” as a proxy for “safe” spaces and “inclusive” as a proxy for “brave” spaces.)

Overall, we noticed that each organization initially establishes exclusive safe spaces to promote confidence and skills for participants to then move more deliberately into more inclusive brave spaces. One of the first phases each organization engaged was building exclusive spaces of demographically similar participants, whether that be across gender (Anyone Can Skate, Skate Center) or language (Skate in School). Within these safe spaces of exclusion, joy, play, and fun are centered as it is pivotal that participants feel a sense of belonging and engagement. The organizer of Skate in School, for instance, explains how the program has quite a lot of “flexibility” and allows participants to “move at their own pace.”

Importantly, these exclusionary spaces often involve direct instruction regarding power structures to help participants recognize, name, and better understand how systems of oppression operate. In this way, these safe spaces of exclusion function to build skills and knowledge—both related to skateboarding and social structures—to help participants know themselves, know about social dynamics, build networks of support and “relational” and “ideational” resources (14), feel connected and a sense of belonging to a group and a place, and imagine and rehearse possibilities to intervene in oppressive circumstances. In other words, these safe spaces of exclusion help lay the groundwork for participants to integrate and impact change within more inclusive “brave” spaces where there is less regulation and safety.

## “Focus day” in Skate in School

Skate in School developed, after careful observation of the demographics of participants, a practice of exclusion called “Focus Day.” As an after-school club, Skate in School draws its participants from the students who attend the school. Though the school serves refugee and immigrant youth from about thirty countries (e.g., Afghanistan, Eritrea, Central American, Mexico), much of the student demographic is Spanish-speaking, and these Spanish-speaking students comprised the main contingency in the skate club. Knowing that students whose home languages were not Spanish desired to participate but were intimidated to do so, River, the main organizer of the club, created a special exclusive occasion for female-identified language minority students who were not Spanish speaking—“a space without Spanish speakers,” as River put it.

Explaining the impact of the event, River said: “We just had a lot of fun. And then they [the girls who participated] started coming to skateboarding. I started seeing like the Afghan girls, and those students from Eritrea and Ethiopia started coming.” In this sense, the move to create an exclusionary “safe” space for the non-Spanish speaking students created an opportunity for them to subsequently participate in the “brave” space of inclusion within the context of the normally operating after-school skate club.

## Anyone Can Skate creating safe spaces within typically public brave spaces

In addition to creating exclusive spaces within an organization, the overall aim of such moves from exclusion to inclusion is so participants can engage brave spaces beyond the organization with a different sense of purpose and possibility. For instance, Anyone Can Skate hosts events at public skateparks exclusively for women-identified and nonbinary participants so that they develop a greater sense of belonging in that specific place that will then facilitate their continued presence past the exclusionary experience. Amy, one of the key organizers of Anyone Can Skate, says, “So the goal is that we try to do this in public spaces so that they're not just in like our coddled space and they're like, ‘Yeah, we did it.’ But we are helping them gain evidence for themselves that they deserve to be here, that they belong here and that they have everything they need.”

In one instance, Amy explains how three young women approached her because they were being harassed at a public skatepark. The organization, then, created an exclusive space with and for these three to do some solution-posing brainstorming sessions for how they might disrupt the harmful activities at the skatepark and raise awareness. Ultimately, the group developed and implemented a set of interventions (including facilitating a “community conversation”) to promote a more inclusive park. In this way, they used the exclusive space to develop skills and a gameplan for how to engage and participate in the more inclusive brave space.

In this way, the brave space is not the organization itself (like in the previous example with Skate in School) but rather public skateparks beyond the purview of the organization. This is similar to how Skate in School uses skateboarding as a way to help students navigate the complex challenges within broader U.S. society as newly-arrived English Language Learners. In other words, the safe spaces of exclusion in the skate organizations are meant to facilitate participants’ engagement in the brave spaces beyond the organization—whether that be a public skatepark or broader U.S. society.

## Exclusion to facilitate allyship in Skate Center

Skate Center has a portion of its programming exclusive to cisgender young men. Building on Ashanti Branch's ideas of “Behind the Mask,” the program is designed to help young men, within that exclusive space, to examine the various ways socially dominant scripts of the “hyper masculinity narrative” show up in and adversely inform their lives. In this way, though cisgender young men are not a marginalized demographic (especially within skateboarding), the recognition here is that exclusive space can engender more authentic explorations of being young men, which is particularly important given how the codes of dominant masculinity often deter exactly this type of emotional introspection and sharing with other men.

The organizer of this activity in Skate Center, Chris, explains: “What they're all about is to have young men talk about things that society does not allow young men to talk about, such as like feeling pain, anger. Crying doesn't make you any less of a man. And so, it's to just reshape this hyper masculinity narrative. And then, so under the teachings of Ashanti Branch, we've then instilled a lot of that with the core skaters that we work with specifically just with the men. And so, we do a lot of ‘Removing the Mask’ workshops with those young men. And helping them navigate that toxic masculinity that's just embedded with it, unfortunately.”

Beyond personal introspection and liberation, such spaces of exclusion can facilitate participants’ understandings of ways they can ally with others in the skateboarding community who do not have the same challenges to access as those with marginalized identities, and, ultimately, help co-create more inclusive, healthy, and safe brave spaces. Amy explains, for instance, how, during exclusive events for women-identified and nonbinary skaters in public skateparks, she will interface with cisgender young men who are upset about the exclusionary nature of the event. She explains how these encounters can be educational and empowering for not just those who are participating but also the young men who are unhappy on the sidelines. She explains that she might offer something like the following to these young men: “Actually the best way you can support is by being an ally—keeping your board in your car tonight and cheering folks on and just stepping back because most of the time and in other spaces, you get to have the space every other night. So, thank you so much for sharing the space with us.”

In thinking of this interaction, the work of Skate Center, by having exclusionary spaces for cisgender men to deconstruct dominant, damaging norms of masculinity, and the work of Anyone Can Skate, by having exclusionary spaces for trans, non-binary, and women-identified skaters, can work synergistically and constitutively to lead toward greater inclusivity beyond each respective organization. A key goal, exemplified through the example of the young women who facilitated community conversations, is to bring together participants *across* these exclusionary spaces to facilitate dialogue and consciousness raising to, ultimately, transform skateboarding and society for more healthy, sustainably welcoming spaces of inclusion. In this sense, the relationship between safe and brave spaces is not linear but can be interdependent, recursive, and iterative, and, thus, can be leveraged consciously in relation to one another.

## Discussion: theories of change

As we examined the practices of these organizations, we found that the concept of brave spaces gave us new ways of thinking about possibilities for cultivating inclusion. Though learning to work within brave spaces is difficult, we believe it is necessary for participants’ sense of agency and dignity, and, ultimately, to create a more inclusive community. Moreover, we are inspired, from this research, to explore more systematically, the ways brave and safe spaces can work in tandem and constitutively to engender inclusion across skateboard spaces.

Across these three organizations, safe spaces of exclusion were used to promote a range of affective and relational experiences and opportunities for fun, engagement, belonging, confidence, trust, safety, friendship, and community building. Alongside this, these organizations used safe spaces of exclusion to engage participants in explicitly learning about, naming, and discussing societal power structures (e.g., heteropatriarchy, toxic masculinity), as well as imagining, planning, and supporting efforts to intervene in oppressive practices and spaces. Amy explains this as having “critical conversations about power” and “building awareness about power differences and social inequities.”

In these ways, these organizations are operating from theories of change that recognize, first, learning as a sociocultural and emotional endeavor—not just about skill acquisition—and that a key facet of promoting participation is attending to the affective experiences of participants. For these organizations, creating exclusive spaces where participants felt like they were welcomed and where they belonged was paramount before anything else. Thus, this attention to the affective might be conceptualized as the *sine qua non* of the theories of change that undergird these organizations’ attempts at social transformation.

A second key facet of these organizations’ theories of change is the understanding that social transformation, especially within potentially fraught “brave” spaces, occurs through an iterative process of learning *about* social systems and acting *upon* them. In this way, these organizations share similarities with the Freirian (15) notion of “praxis,” which speaks to participants engaging in a cyclical movement from “active reflection,” in which they come together to name and learn about social systems and power dynamics, and “reflective action,” whereby they act upon their environment to transform it based on their understandings of oppression and liberation.

For these organizations, this movement between active reflection and reflective action was facilitated by the pedagogical practice of establishing safe spaces of exclusion to support intervention in brave spaces of inclusion. This movement between safe spaces of exclusion and brave spaces of inclusion is critical as possibilities for true inclusion must move beyond open access: it's not just about opening access but critiquing and transforming systems of power; without this, there will not be inclusive participation on an *everyday basis*.

## Data Availability

The datasets presented in this article are not readily available because the full dataset is available only to the PIs for the research project. Requests to access the datasets should be directed to petroner@missouri.edu.

## Footnotes

^1^Names of all participants and organizations are pseudonyms.
